# An Improved Multi-Sensor Fusion Navigation Algorithm Based on the Factor Graph

**DOI:** 10.3390/s17030641

**Published:** 2017-03-21

**Authors:** Qinghua Zeng, Weina Chen, Jianye Liu, Huizhe Wang

**Affiliations:** 1 College of Automation Engineering, Nanjing University of Aeronautics and Astronautics, Nanjing 211100, China; ljyac@nuaa.edu.cn (J.L.); hzwang@nuaa.edu.cn (H.W.); 2 Satellite Communication and Navigation Collaborative Innovation Center, Nanjing 211100, China

**Keywords:** micro unmanned aerial vehicle, multi-sensor information fusion, factor graph, probability density

## Abstract

An integrated navigation system coupled with additional sensors can be used in the Micro Unmanned Aerial Vehicle (MUAV) applications because the multi-sensor information is redundant and complementary, which can markedly improve the system accuracy. How to deal with the information gathered from different sensors efficiently is an important problem. The fact that different sensors provide measurements asynchronously may complicate the processing of these measurements. In addition, the output signals of some sensors appear to have a non-linear character. In order to incorporate these measurements and calculate a navigation solution in real time, the multi-sensor fusion algorithm based on factor graph is proposed. The global optimum solution is factorized according to the chain structure of the factor graph, which allows for a more general form of the conditional probability density. It can convert the fusion matter into connecting factors defined by these measurements to the graph without considering the relationship between the sensor update frequency and the fusion period. An experimental MUAV system has been built and some experiments have been performed to prove the effectiveness of the proposed method.

## 1. Introduction

In recent years, the use of low-cost miniature unmanned aerial vehicles (MUAVs) for civilian applications has evolved from imagination to actual implementation. Systems have been designed for environmental monitoring, search and rescue, mapping, and mining and post seismic emergency management [1,2,3,4,5]. In order to obtain an acceptable cost/benefit ratio of these systems, there have been many techniques to reduce the cost including the aspects of the sensor, the platform, and the algorithm [6].

However, cost reduction of the sensor can lead to lower stability and accuracy, which is directly related to the reliability of the system. Thus, dependence on a single sensor cannot satisfy the demand and makes it difficult to obtain the accurate response to external environment. Through integrated usage of multiple-source information, people can get more objective and intrinsic knowledge of a certain target [7]. As a matter of fact, multi-sensor integrated navigation is becoming a hot area of research. Many studies focus on the integration of the GPS/INS system with additional sensors such as magnetometers, cameras, ultrasonic sensors, laser scanners, barometers, or earth/sun/star sensors [8]. The combination scheme can make use of all information from these sensors and overcome their limitations, which allows for the provision of reliable navigation solutions [9].

In order to achieve the optimal solution in the integrated navigation, the fundamental problem is information fusion [10]. In general, multi-sensor information fusion is a form of sensor integration, which involves combining the outputs of different sensor systems to obtain a better estimate of what they are sensing according to some fusion rules [11,12]. Nowadays, multi-sensor data fusion has attracted widespread attention, its theory and methods have been applied to a lot of research fields [13,14]. The conventional approach employed in reference [15] uses the centralized Kalman filtering method which processes all of the measurements at a central processor and provide a global optimal estimate. The advantage of this method is that it involves minimal information loss. However, the centralized filter can result in a large computational burden and poor fault tolerance due to overloading of the filter with too many data [16]. To deal with these problems, decentralized information fusion methods [17,18,19] have been developed in fields such as distributed control systems and integrated navigation. In decentralized fusion, a processor at the center of the network is used to manage all nodes. The decentralized fusion algorithms are based on the global optimality criterion, which has reasonable fusion accuracy, good fault-tolerance, and lower computing effort requirements. Among them, the federated Kalman filtering algorithm [20] and the parallel Kalman filtering algorithm [21] are representative. However, if the dimensions of the measurement vector are too high, the order of the measurement noise covariance matrix complicates the calculation of an inverse. Dempster-Shafer theory (DST) is an efficient method to deal with incomplete data coming from different information sources in data fusion [22]. It does not rely on any prior knowledge of the probability distribution. Thus, how to combine the information from different sources, which may be conflicting, becomes a challenging problem in complex decision making. The method referred in reference [23] focuses on the research on sequential decentralized filters. This method saves a great deal of computing time, especially for the systems with some measurement delays within one sampling period, where the updates are operated without waiting for all the data [24]. However, in the practical applications, an MUAV may be equipped with a set of multi-rate sensors, and these sensors generally operate at different frequencies. So it may complicate the processing of these measurements [25]. In addition, the output signals of some sensors appear non-linear in character. How to incorporate these measurements and calculate the optimal navigation solution by fusing all the available information sources has not been fully investigated. 

In this paper, based on the principle of the probability graph model, a new approach for information fusion in multi-sensor integrated navigation systems is proposed. At first, the structure of the micro unmanned aerial vehicle system is described and the model for the navigation system is built. Then the details about information fusion method based on the factor graph are presented. An experimental system has been built and experimental results have been designed to prove the effectiveness of the proposed method.

## 2. System Overview

In this section, the hardware structure of the MUAV system is described. The MUAV has the characteristics of small size and light weight. A multi-sensor navigation system plays a key role in the MUAV system. According to the characteristics of different navigation sensors, the hardware is designed to enhance the expandability of the system. Considering the flexibility and high efficiency of the hardware structure, dual-redundant sensors are used in order to improve performance of the navigation system. The redundant sensors are beneficial for monitoring the health of each sensor if one sensor fails to work. The hardware also consists of the Cortex-M4 core ARM processor, RC signal processing, motor drive circuit, SD card data storage, and the communication interface.

The MUAV is equipped with a variety of sensors, including the GPS receiver, optic flow sensor, sonar sensor, Xbee wireless communication module, electron speed regulator, and motors, etc. Different sensors and their performance are shown as follows.

- MPU-6000 inertial sensor, including a three-axis MEMS gyroscope and a three-axis accelerometer.
- HMC5983 magnetometer enables 1° to 2° compass heading accuracy with temperature compensation.
- MS5611 barometer module, with an altitude resolution of 0.1 m.
- URM37 sonar module provides 0.04 m–5 m non-contact measurement function, the ranging accuracy can reach to 1 cm.
- Ublox LEA 6H GPS receiver, with the position accuracy of 2 m.
- Optical flow sensor processing the pixel resolution of 752 × 480 at 120 (indoor) to 250 Hz (outdoor).

**Figure 1:**
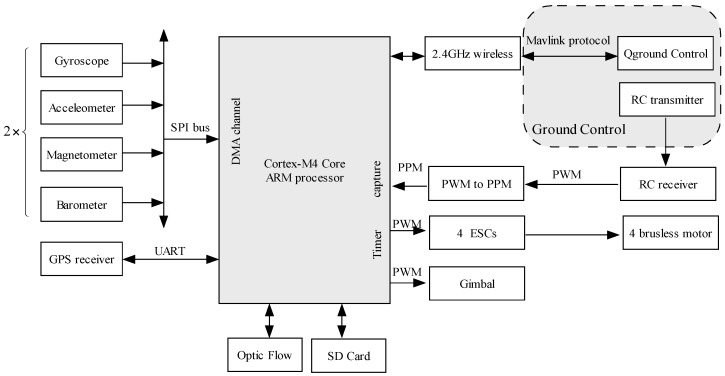
System hardware structure of the MUAV.

The ground control station used is the advanced open-source ground Control Station software, which can realize 2/3D aerial maps with drag-and-drop waypoints. The SPI bus is used for interfacing the sensor to our microcontroller which makes the measurements available to our ground control station. The hardware structure of the MUAV is shown in Figure 1.

## 3. System Model for the Navigation System

In order to accomplish the navigation, the system equations of the system can be established. In this system, the local North-East-Down frame is selected as navigation frame.

### 3.1. State Model of the System

The state variable of the micro unmanned aerial vehicle system can be chosen as follows.

(1) $$X(t)=\left[\begin{array}{ccccc} p^{T} & v^{T} & q^{T} & \nabla_{a}^{T} & \nabla_{g}^{T} \end{array}\right]$$

where $p={\left[\begin{array}{ccc} x & y & z \end{array}\right]}^{T}$ represents the relative position vector to the desired hover position navigation position, $v={\left[\begin{array}{ccc} v_{x} & v_{y} & v_{z} \end{array}\right]}^{T}$ represents the velocity vector, $q={\left[\begin{array}{cccc} q_{0} & q_{1} & q_{2} & q_{3} \end{array}\right]}^{T}$ represents the attitude quaternion, $\nabla_{a}={\left[\begin{array}{ccc} \nabla_{a_{x}} & \nabla_{a_{y}} & \nabla_{a_{z}} \end{array}\right]}^{T}$ is the acceleration random walk term, and $\nabla_{g}={\left[\begin{array}{ccc} \nabla_{g_{x}} & \nabla_{g_{y}} & \nabla_{g_{z}} \end{array}\right]}^{T}$ is the gyro random walk term.

The continuous time kinematic navigation equations are given by:

(2) $$\begin{array}{l} \dot{p}=v\\ \dot{v}=C_{b}^{n}\cdot ({\tilde{f}}^{b}-\nabla_{a}-\omega_{a})+{\left[\begin{array}{ccc} 0 & 0 & 1 \end{array}\right]}^{T}g\\ \dot{q}=\frac{1}{2}q⊗({\tilde{w}}_{ib}^{b}-\nabla_{g}-\omega_{g})\\ {\dot{\nabla }}_{a}=\omega_{b_{a}}\\ {\dot{\nabla }}_{g}=\omega_{b_{g}} \end{array}$$

where, $\left[\begin{array}{cccccc} \omega_{p}^{T} & \omega_{v}^{T} & \omega_{b_{a}}^{T} & \omega_{b_{g}}^{T} & \omega_{a}^{T} & \omega_{g}^{T} \end{array}\right]$ are the gaussian white noises.

### 3.2. Measurement Model of the System

In the MUAV navigation system, the measurement model should be established below.

#### 3.2.1. GPS Measurement Equation

The GPS receiver measures the MUAV’s velocity and position. Hence, the GPS measurement equation is described as Equation (3).

(3) $$\begin{array}{l} p_{gps}=p+\omega_{p}^{gps}\\ v_{gps}=v+\omega_{v}^{gps} \end{array}$$

where $p_{gps},v_{gps}$ are measurements of the GPS receiver; and $\omega_{p}^{gps}$, $\omega_{v}^{gps}$ are the gauss stochastic measurement noise terms.

#### 3.2.2. Barometric Altimeter Measurement Equation

The barometric altimeter is used to measure barometric pressure and translate it into an output of voltage ranging from zero to five volts. The barometric errors vary in accordance with the environment. The observation model of the barometer sensor is given by

(4) $$h_{pres}=h+\omega_{pres}$$

where $h_{pres}$ is the altitude measured by the barometer, h is the true altitude, and $\omega_{pres}$ is the white noise of the barometer.

The height measured by the barometric altimeter is relative to the sea level, while the height measured by the GPS receiver is based on the WGS84 coordinate system. There are some differences between them. In practical application, the height has to be converted to a uniform standard and then fused. The sea level parameter correction method is often used to compensate the barometric altimeter bias. Actual sea level parameters can be calculated by using the atmospheric parameters of the location when the altitude is known. With the modified sea-level parameters, the barometric altimeter bias will be compensated.

#### 3.2.3. Magnetometer Measurement Equation

The magnetometer is one of the common sensors used in MUAV. It is lightweight and reliable. It can provide the direction of the magnetic field, which can be used as aiding information. The magnetometer output $m^{b}$ is not influenced by the maneuvering acceleration. Thus, the well-compensated magnetometer data are used to measure the attitude matrix $C_{n}^{b}$ to enhance the accuracy of the attitude estimation. The magnetometer measurement equation is described as Equation (5).

(5) $$m^{b}=C_{n}^{b}\cdot m^{n}+\omega_{m}$$

(6) $$C_{b}^{n}=\left[\begin{array}{ccc} q_{0}^{2}+q_{1}^{2}-q_{2}^{2}-q_{3}^{2} & 2(q_{1}q_{2}-q_{0}q_{3}) & 2(q_{1}q_{3}+q_{0}q_{2})\\ 2(q_{1}q_{2}+q_{0}q_{3}) & q_{0}^{2}-q_{1}^{2}+q_{2}^{2}-q_{3}^{2} & 2(q_{2}q_{3}-q_{0}q_{1})\\ 2(q_{1}q_{3}-q_{0}q_{2}) & 2(q_{2}q_{3}+q_{0}q_{1}) & q_{0}^{2}-q_{1}^{2}-q_{2}^{2}+q_{3}^{2} \end{array}\right]$$

where $m^{n}$ is the magnet vector in navigation frame. Supposing the local magnetic declination and magnetic inclination are $\theta_{dec}$ and $\theta_{inc}$ respectively, then $m^{n}=\left[\begin{array}{c} \cos\theta_{inc}\cos\theta_{dec}\\ \cos\theta_{inc}\sin\theta_{dec}\\ -\sin\theta_{inc} \end{array}\right]$.

#### 3.2.4. Optical Flow Measurement Equation

The downward-looking visual system is called the PX4-FLOW sensor, which is an open source embedded metric optical flow CMOS camera designed for indoor and outdoor applications. The raw pixel movement data are used to calculate the horizontal velocity. The observation model of the optic-flow sensor is given by

(7) $$v_{optic}=kv+\omega_{optic}$$

where $v_{optic}$ is the velocity acquired by the optic-flow sensor, v is the true horizontal velocity, $\omega_{optic}$ is the white noise of optic-flow observation, and k represents the coefficient determined by the camera focal length and altitude of the MUAV.

#### 3.2.5. Sonar Measurement Equation

The sonar sensor and the barometric altimeter are often used to measure the altitude in the MUAV system. Because the sonar sensor has advantages of high precision, narrow measuring range and good stability, it can also meet the demand of indoor or low altitude flight. The barometric altimeter can be easily affected by environment, but it has a wide measuring range. So it can be used in the outdoor or high altitude flight. In some specific environments, these two kinds of sensors can be used in combination to acquire high reliability. The sonar measurement equation is described as Equation (8).

(8) y=kx+b

where y is the output of the sonar, x is the true distance, k represents the coefficient of the first order, and b is the fixed distance error. The error coefficients k and b are acquired by laboratory calibration tests.

## 4. Information Fusion Method Based on the Factor Graph

The factor graph is a graphic model that encodes the conditional probability density among unknown variable nodes and the received measurements. The global optimum solution is factorized according to the chain structure of the factor graph, which allows for a more general form of the conditional probability density. It can convert the fusion matter into connecting factors defined by these measurements to the factor graph without considering the relationship between the sensor update frequency and the fusion period. 

### 4.1. Factor Graph Formulations

The probability graph model is a kind of graph model which can express the joint probability distribution of random variables. The factor graph is a bipartite graph representing the factorization structure of a multivariate function into a product of functions (factors), each involving only a subset of the variables [26]. There are two kinds of nodes in the graph. The variable node X represents the variable of the global multivariate function, and the factor node F represents the factor in the factorization. The variable node is connected to a factor node by an edge E [27]. The factor graph is defined as

(9) G=(F,X,E)

where $X_{i}\subseteq \{x_{1},x_{2},\cdots ,x_{m}\}$ is the subset of the variable nodes $x_{j}(j=1,2,...,m)$, m is the number of the variable node, $F=\left\{f_{1}(X_{1}),f_{2}(X_{2})...,f_{n}(X_{n})\right\}$ represents a set of the factor nodes $f_{i}(X_{i})(i=1,2,...,n)$, and n is the number of the factor node. Edges $E=\left\{e_{ij}\right\}$ denotes the set of links of the factor graph, which can exist only between factor nodes and variable nodes. The factor graph G defines one factorization of the function as

(10) $$g(X)=\prod_{i}f_{i}(X_{i})$$

**Figure 2:**
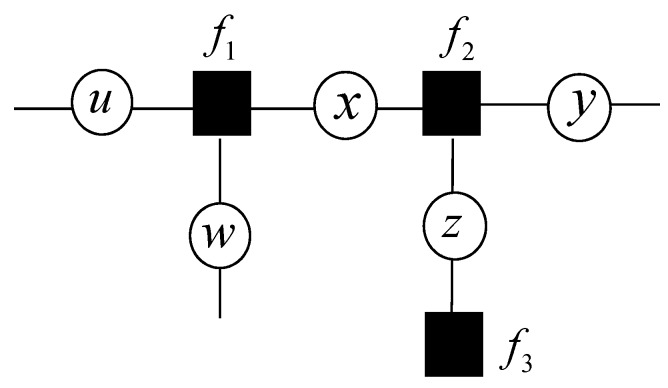
An example of a factor graph.

A factor graph can be used as a probabilistic graphical model which represents a joint probability mass function of random variables. Each variable node represents a set of the random variables. Each factor represents the joint probability function of a subset of random variables, which is shown as a box or a circle. For example, Figure 2 can be factored as

(11) $$g(u,w,x,y,z)=f_{1}(u,w,x)f_{2}(x,y,z)f_{3}(z)$$

The factor vertex *f*_1_, *f*_2_, *f*_3_ are the local functions. Due to the variable vertex *u*, *w*, *x* are connected to *f*_1_, the factor vertex *f*_1_ involves the independent variables *u*, *w*, *x*. In a similar way, the factor vertex *f*_2_ involves the independent variables *x*, *y*, *z*. The factor vertex *f*_3_ involves the independent variable *z*. 

### 4.2. Fusion Algorithm with the Factor Graph

For our application, the factor graph framework model is established for the MUAV navigation system. This graph-based optimization algorithm is particularly developed to solve the optimization problem for the current state prediction with asynchronous multi-sensor data. The main purpose is to calculate the best navigation solution by using the available information sources.

The state variable of the MUAV system X is described in Section 3.1. $X_{k}\in R^{n}$ is the state variable in the current time $t_{k}$. Given the initial distribution $p(X_{0})$, $X_{k}$ can propagate with the probability density $p(X_{k}|X_{k+1})$. $Z_{k}\in R^{m}$ represents the independent measurement in the current time $t_{k}$. The Bayesian estimation contains two parts, the prediction and the update. The prediction concerns the prior density of the state with the state model. The update process is to modify the prior density obtained by the previous step by using the newest measurement in order to obtain an a posteriori probability density $p(X_{0:k}|Z_{1:k})$. Our goal is to solve the a posteriori probability density in the navigation system [28].

Given the observations up to time k, the global conditional joint probability density function $p(X_{0:k}|Z_{1:k})$ for the state vector $X_{0:k}$ is the marginal function. According to the Bayes formula, $p(X_{0:k}|Z_{1:k})$ can be factorized as:

(12) $$\begin{array}{cc} p(X_{0:k}|Z_{1:k}) & =\frac{p(Z_{k}|X_{k})p(X_{k}|X_{k-1})}{p(Z_{k}|Z_{1:k-1})}\cdot p(X_{0:k-1}|Z_{1:k-1})\\ & =\frac{p(Z_{k}|X_{k})p(X_{k}|X_{k-1})}{p(Z_{k}|Z_{1:k-1})}\cdot \frac{p(Z_{k-1}|X_{k-1})p(X_{k-1}|X_{k-2})}{p(Z_{k-1}|Z_{1:k-2})}\\ & \;\cdot p(X_{0:k-2}|Z_{1:k-2})\\ & =\frac{p(Z_{k}|X_{k})p(X_{k}|X_{k-1})}{p(Z_{k}|Z_{1:k-1})}\cdot \frac{p(Z_{k-1}|X_{k-1})p(X_{k-1}|X_{k-2})}{p(Z_{k-1}|Z_{1:k-2})}\cdot ...\\ & \;\cdot \frac{p(Z_{1}|X_{1})p(X_{1}|X_{0})}{p(Z_{1})}\cdot p(X_{0})\\ & =\prod_{i=1}^{k}\frac{p(Z_{i}|X_{i})p(X_{i}|X_{i-1})}{p(Z_{i}|Z_{1:i-1})}p(X_{0}) \end{array}$$

where $p(X_{0})$ represents all of the available prior information. This global conditional probability density function is proportional to the likelihood probability density and the state transition prior probability in the numerator.

(13) $$\frac{p(Z_{i}|X_{i})p(X_{i}|X_{i-1})}{p(Z_{i}|Z_{1:i-1})}p(X_{0})\propto p(Z_{i}|X_{i})p(X_{i}|X_{i-1})$$

According to the maximum a posteriori criterion, the state variable with the maximum a posteriori probability density is considered as the estimation.

(14) $${\hat{X}}_{i}^{MAP}=\arg\max p(X_{i}|Z_{i})$$

Equation (12) can be written as

(15) $$\prod_{i=1}^{k}\frac{p(Z_{i}|X_{i})p(X_{i}|X_{i-1})}{p(Z_{i}|Z_{1:i-1})}p(X_{0})\propto \prod_{i=1}^{k}p(Z_{i}|X_{i})p(X_{i}|X_{i-1})$$

To calculate the ${\hat{X}}_{i}^{MAP}$ in Equation (14), the right side of Equation (15) will be maximized. For Gaussian noise distributions, the probability density $p(Z_{i}|X_{i})$can be calculated as:

(16) $$p(Z_{i}|X_{i})=\frac{1}{{\left(2\pi \right)}^{k/2}{\left(R\right)}^{1/2}}\exp\left\{-\frac{1}{2}{\left[Z_{i}-h_{i}(X_{i})\right]}^{T}W^{-1}[Z_{i}-h_{i}(X_{i})]\right\}$$

where W is the variance matrix, $h_{i}()$ is measurement function, and $Z_{i}$ is the actual measurement. For the above-mentioned reasons, Equation (17) should be minimized as the following function

(17) $$J={\left[Z_{i}-h_{i}(X_{i})\right]}^{T}W^{-1}[Z_{i}-h_{i}(X_{i})]=\min$$

For linear measurement, the optimal solution ${\hat{X}}_{i}$ can be solved by using the method of seeking extreme. For nonlinear measurement, it can be solved by using Newton iteration method. When adding the new factor node $Z_{x,i}$ to the graph, the measurements of different sensors are used to calculate the optimal solution.

By the similar method, the probability density $p(X_{i}|X_{i-1})$ can be calculated as the same way. The probability density $p(X_{i}|X_{i-1})$ can be calculated as

(18) $$p(X_{i}|X_{i-1})=\frac{1}{{\left(2\pi \right)}^{k/2}{\left(P\right)}^{1/2}}\exp\left\{-\frac{1}{2}{\left[X_{i}-{\hat{X}}_{i}(Z_{i})\right]}^{T}P^{-1}[X_{i}-{\hat{X}}_{i}(Z_{i})]\right\}$$

where *P* is the variance matrix. Equation (19) should be minimized as the following function:

(19) $$J={\left[X_{i}-{\hat{X}}_{i}(Z_{i})\right]}^{T}P^{-1}[X_{i}-{\hat{X}}_{i}(Z_{i})]=\min$$

**Figure 3:**
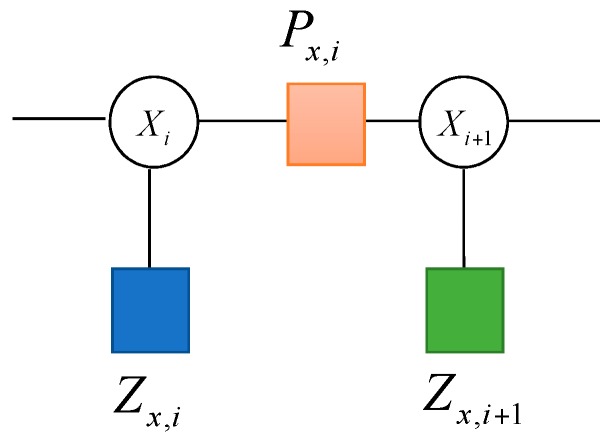
Factor graph representations of the state variable and the measurement.

The estimation of the state ${\hat{X}}_{i/i-1}$ can be solved by state vectors propagating. When adding the new variable $X_{i+1}$ to the graph, the IMU measurement is used to calculate the state transition matrix to predict the values for $X_{i+1}$. In the factor graph. Let f() represent the local functions of the probability density function. Equation (12) can be written as

(20) $$\prod_{i=1}^{k}\frac{p(Z_{i}|X_{i})p(X_{i}|X_{i-1})}{p(Z_{i}|Z_{1:i-1})}p(X_{0})\propto \prod_{i=1}^{k}f(Z_{i}|X_{i})f(X_{i}|X_{i-1})$$

where $f(Z_{i}|X_{i})$ and $f(X_{i}|X_{i-1})$ are the local functions associated with the factor nodes $P_{x,i}$ and $Z_{x,i}$ in Figure 3, respectively.

The factor graph model of the navigation system is dominated by the two local functions $f(Z_{i}|X_{i})$ and $f(X_{i}|X_{i-1})$. By connecting the variable nodes associated with each function to the factor node, the factor graph can be constructed. The factor graph method allows for a more general form of the conditional probability density.

### 4.3. Factor Graph Modeling

**Figure 4:**
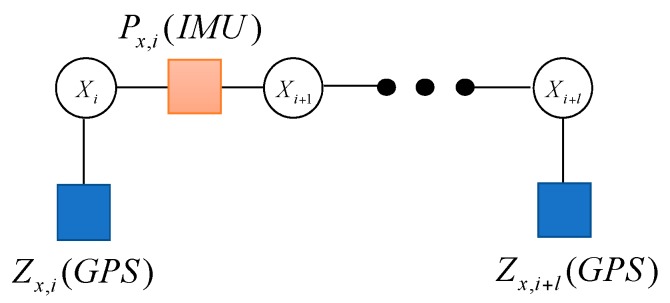
The factor graph containing the GPS measurement.

Not all of the sensors can be updated at the same time. In general, the frequencies of the IMU measurements are very high, which are different from other sensors. For example, the update frequency of GPS measurement is slower than IMU, which can be described in Figure 4.

Variable node $X_{i}$ represents the state variable of the system. $f_{x,i}(IMU)$ denotes the updating of the variable node with the IMU measurement $P_{x,i}(IMU)$. The factor IMU is defined as the following form

(21) $$f_{x,i}(IMU)=p(X_{i+1}|X_{i})$$

It connects two different variable nodes $X_{i}$ and $X_{i+1}$ in $t_{i}$ and $t_{i+1}$ moments, respectively. The IMU measurement is used to calculate the state transition matrix to predict ${\hat{X}}_{i+1/i}$ by state vector propagation.

On the other hand, $f_{x,i}(GPS)$ denotes the factor node when the system receives the measurement $Z_{x,i}(GPS)$. The factor GPS is defined as the following form

(22) $$f_{x,i}(GPS)=p(Z_{i}^{GPS}|X_{i})$$

The estimation of $X_{k}$ can be solved by using the method of seeking extreme through the probability density function $p(Z_{i}^{GPS}|X_{k})$. It can be calculated as Equations (16) and (17). With this model, we can calculate the minimization of the local functions $f(Z_{i}|X_{i})$ and $f(X_{i+1}|X_{i})$ to acquire the optimal state at $t_{i}$ moment.

**Figure 5:**
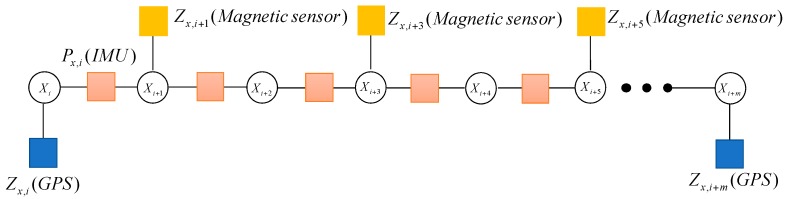
The factor graph containing GPS and magnetic measurement.

When the system receives the magnetic measurement $Z_{x,i+1}(Magnetic\;sensor)$, the factor node $f_{x,i+1}(Magnetic\;sensor)$ will be added into the graph. Because the update frequency of the magnetic sensor is faster than that of GPS, the magnetic sensor nodes appear many times during the update of GPS. It is described in Figure 5.

**Figure 6:**
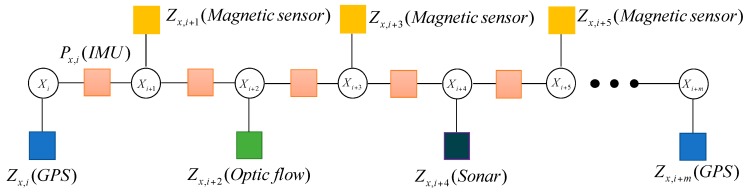
The multi-sensor fusion framework based on factor graph.

The update frequencies of different sensors in the MUAV are different. In a similar way, the factor graph model describes the characteristics of the MUAV navigation system, which are shown in Figure 6.

This model enables us to handle situations where different sensors provide measurements at different times and at different time intervals. When the measurement equation of the new sensor is nonlinear, the method can simply add new factors to the graph. If a sensor becomes unavailable due to various reasons, the system simply stops adding the associated factors to the graph. There is no need for a special procedure.

To implement the factor graph method, the steps are introduced as follows:

Step 1: Set the initial parameters and define a state-space vector. New factors $f_{new}=\left\{\right\}$ and new variables $X_{new}=\left\{\right\}$ are initialized. The probability density function $p(X_{0})$ should be set up according to the parameters of the system.
Step 2: When the system receives the IMU measurement ${\tilde{f}}^{b},{\tilde{w}}_{ib}^{b}$, at $t_{i}$ moment, the factor node $P_{x,i}(IMU)$ will be added into the graph. It connects two different variable nodes $X_{i}$ and $X_{i+1}$ in $t_{i}$ and $t_{i+1}$ moments, respectively. The IMU measurement is used to calculate the state transition matrix to predict ${\hat{X}}_{i+1/i}$ by state vector propagation.
  - Position is calculated by $p_{i+1}=p_{i}+v_{i}\cdot \Delta t$;
  - Velocity is calculated by $v_{i+1}=v_{i}+[C_{b}^{n}\cdot ({\tilde{f}}^{b}-\nabla_{a}-\omega_{a})+{\left[\begin{array}{ccc} 0 & 0 & 1 \end{array}\right]}^{T}g]\cdot \Delta t$;
  - Attitude is calculated by $q_{i+1}=q_{i}+[\frac{1}{2}q_{i}⊗({\tilde{w}}_{ib}^{b}-\nabla_{g}-\omega_{g})]\cdot \Delta t$.
Step 3: Add $X_{i+1}=\left[\begin{array}{ccccc} {p_{i+1}}^{T} & {v_{i+1}}^{T} & {q_{i+1}}^{T} & \nabla_{a}^{T} & \nabla_{g}^{T} \end{array}\right]$ to $X_{new}=\left\{\right\}$;
Step 4: When the system receives the measurement $Z_{x,k}$ (magnetic, GPS, sonar or optic flow, etc.) at $t_{k}$ moment, the factor node $f_{x,k}$(magnetic, GPS, sonar or optic flow, etc.) will be added into the graph. Add $f_{x,k}$ to $f_{new}=\left\{\right\}$.
Step 5: The optimization problem encoded by the factor graph is solved by Gauss–Newton iterations. Z is the set of all measurements, and X represents the set of all variables. $\hat{X}$ is an initial estimate of X. According to Equation (17), the increment ΔX needs to be calculated, which should satisfy Equation (23).
  (23) $$\underset{\Delta X}{\arg\min}{\left\|J(\hat{X})\Delta X-r(\hat{X})\right\|}^{2}$$
  where $J(\hat{X})$ is the Jacobian matrix and $r(\hat{X})$ is the residual of all measurements.

**Figure 7:**
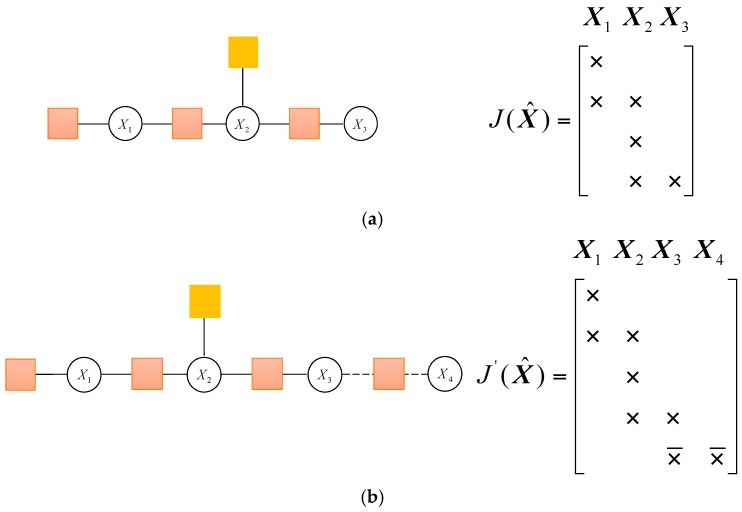
The factor graph and the associated Jacobian matrix in two moments. (**a**) The factor graph and the associated Jacobian matrix; (**b**) The factor graph and the associated Jacobian matrix when adding new factor node.

Calculating the increment ΔX requires factoring the Jacobian matrix into an equivalent upper triangular form, such as QR factorization. Once the increment ΔX is calculated, the new estimate $\hat{X}+\Delta X$ can be obtained. It is set to be the initial estimate in the next iteration. For an example of the system, the factor graph in Figure 7a contains the following four factors which are expressed in the box. The system in Figure 7b adds a new factor, a block row is added in the Jacobian matrix, which is denoted as $\bar{\times }$.

The square root information matrix *R* in Figure 7a is as follows, which is obtained by QR factorization of the Jacobian matrix:

(24) $$\begin{array}{cc} & \begin{array}{ccc} X_{1} & X_{2} & X_{3} \end{array}\\ R= & \left[\begin{array}{ccc} \times & \times & \\ & \times & \times \\ & & \times \end{array}\right] \end{array}$$

When the new factor node is added in Figure 7b, the corresponding square root information matrix *R*’ can be changed as follows:

(25) $$\begin{array}{cc} & \begin{array}{cccc} X_{1} & X_{2} & X_{3} & X_{4} \end{array}\\ R\prime = & \left[\begin{array}{cccc} \times & \times & & \\ & \times & \times & \\ & & \bar{\times } & \bar{\times }\\ & & & \bar{\times } \end{array}\right] \end{array}$$

The modified value, which is different from *R*, is denoted as $\bar{\times }$. It can be seen that the first two block rows remain unchanged. So, there is no need to recalculate these values.

Step 6: Determine whether the new measurement is received by the system. Return to Step 2.

## 5. Experiment and Discussion

### 5.1. Simulation and Analysis

In order to verify the performance of the factor graph method proposed in this paper, the simulation and analysis are carried out with different methods. In the simulation, a typical flight trajectory has been designed. We assume a MUAV equipped with many sensors, including IMU, GPS receiver, magnetometer, and barometer. IMU sensors produce measurements at a high rate while other sensors generate measurements at lower rates. The flight time is 600 s, and the initial position is [106.5°, 29.53°, 25 m]. Parameters of navigation sensors are shown in Table 1.

**Figure 8:**
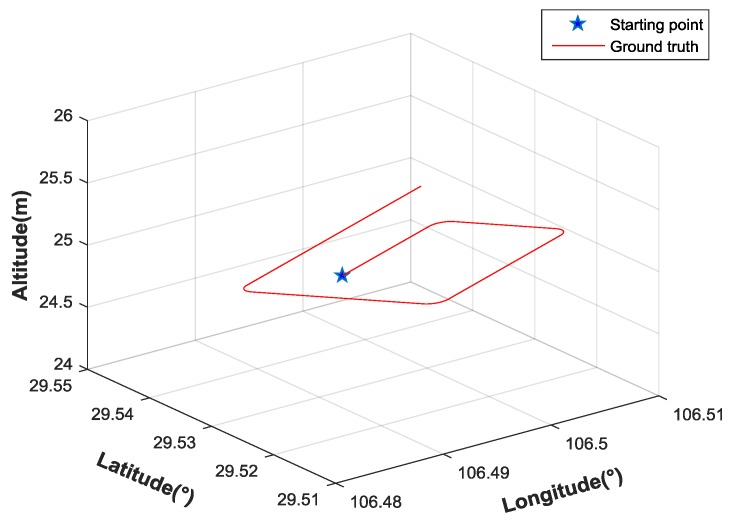
Flight track of the MUAV in the simulation.

The extended Kalman filter (EKF) has been used widely in integrated navigation systems, which linearizes all nonlinear models. In the traditional EKF algorithm, the state propagates with the IMU update rate, and performs a measurement update whenever the measurements are available. The factor graph method is compared with traditional EKF method in the simulation. The flight track is shown in Figure 8. The five-pointed star represents the starting point. The flight altitude remains the same.

**Figure 9:**
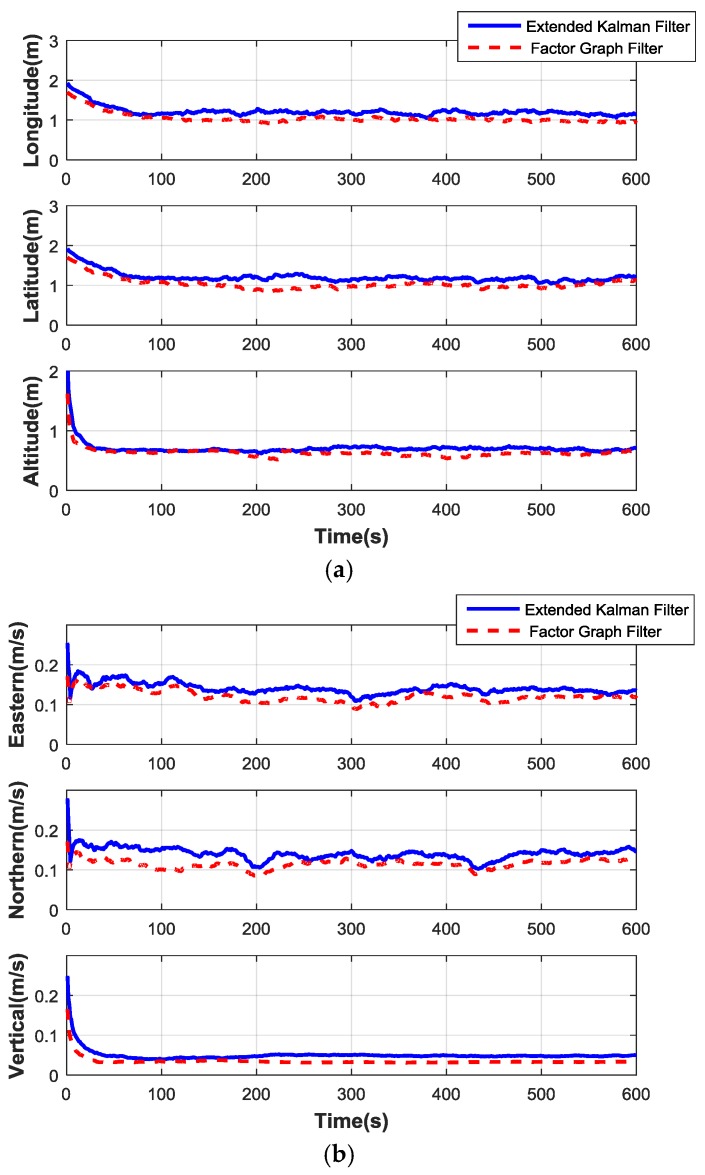
Comparison curves of two filter methods. (**a**) Position error contrast curves; (**b**) Velocity error contrast curves.

Monte-Carlo simulations have been performed and the RMSE performances are used to compare the accuracy of different algorithms. After 100 Monte Carlo simulations, the average RMSE of both methods are given in Table 2. Compared with the ground truth data from the flight track, Figure 9a,b illustrates the average RMSE of the position error and the velocity error generated by these two algorithms, respectively.

From the above simulation results, the average RMSE performances of the EKF and the factor graph filter are compared in Table 2. The improvement of the factor graph filter is obvious. The corresponding accuracy in the velocity and position of the factor graph method improve as well. 

### 5.2. MUAV Flight Test and Results

Outdoor autonomous flight tests on real data have been carried out in a playground. All of the sensors described in Section 2 are mounted on the vehicle. To evaluate the navigation performance, a more precise navigation system which has a differential GPS/INS system on the autopilot board is used as the reference system. For the multi-sensor navigation, GPS positioning is used in single point mode. We make use of the ARM processor with highly optimized C language to ensure real-time performance. The data are captured from the autopilot board of the MUAV during the flight. The total flight time was about 500 s.

**Figure 10:**
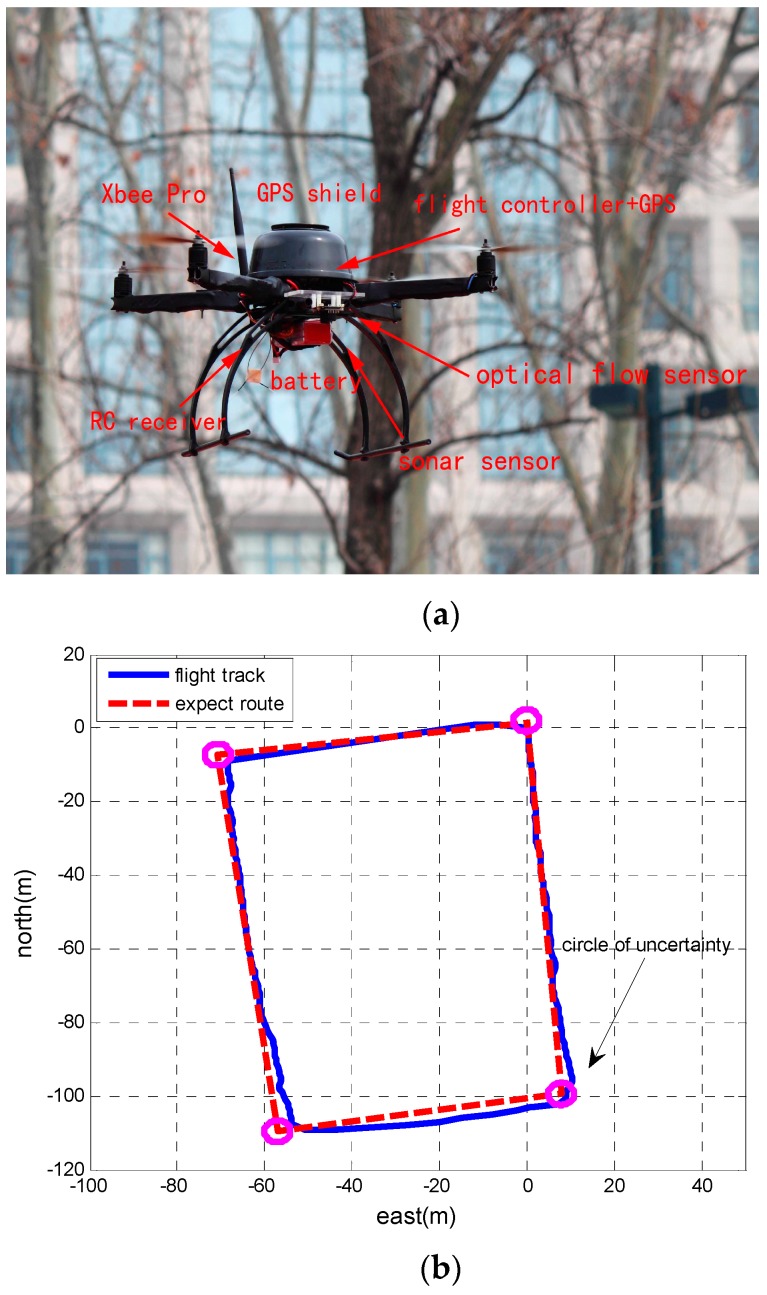
Square shaped outdoor trajectory in the flight experiment of the MUAV. (**a**) Test flight experiment of the MUAV; (**b**) Square shaped trajectory.

The MUAV system in the test flight experiment is shown in Figure 10a. Autonomous flights include takeoff, waypoint, returning, and landing modes. The horizontal flight track compared with the expected route is presented in Figure 10b. System dynamic parameters are shown in Table 3.

**Figure 11:**
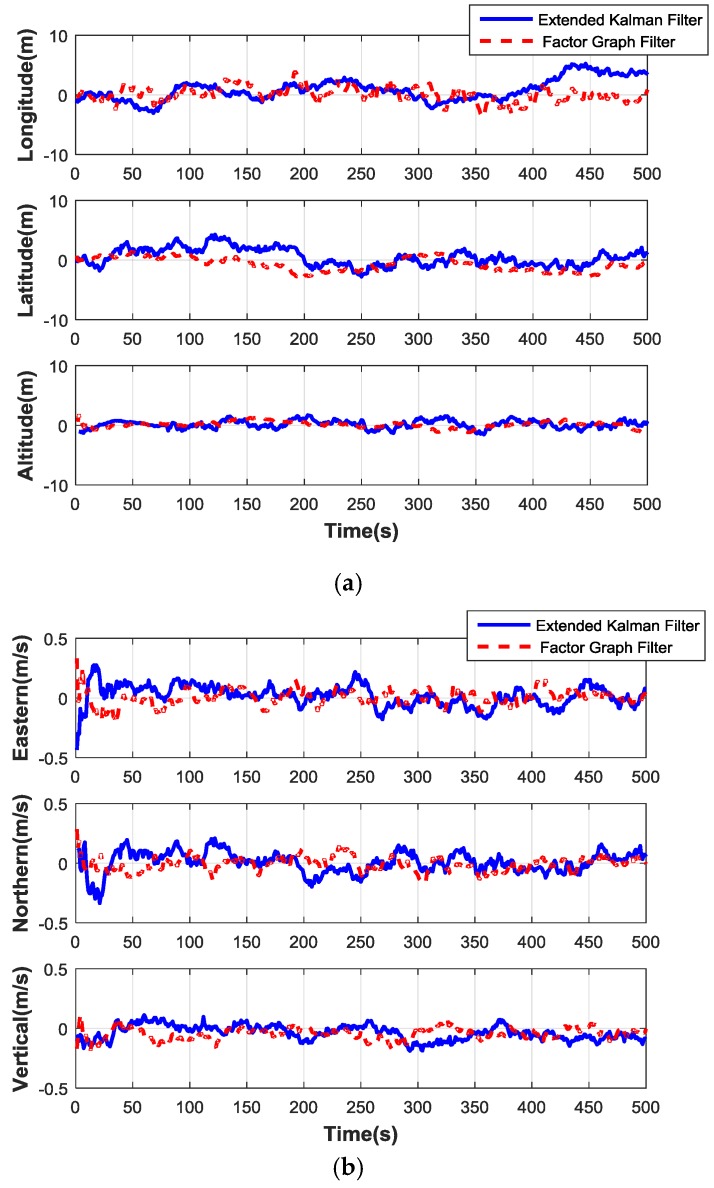
Comparison curves of two filter methods. (**a**) Position error contrast curves. (**b**) Velocity error contrast curves.

The expected route with four waypoints is the pre-set track which is uploaded in the MUAV system before taking off. The circles of the four corners in the square represent the waypoints, and the default waypoint radius is configured to 3 m. This means that if the vehicle reaches the waypoint within the default waypoint radius, it will turn to the next waypoint. The flight track is obtained by the data of the MUAV navigation system. It can be seen that the vehicle can fly along the expected route. Figure 11a,b illustrates the performance of the position error and the velocity error generated by these two filters.

The position and velocity errors of the factor graph filter and EKF are compared in Table 4. It can be seen that the factor graph method has higher precision than EKF. The RMSE results reduce to less than 80%. Better results of the system by using the factor graph filter method can be obtained, and the accuracy level can meet the requirements of the MUAV navigation system.

## 6. Conclusions

In this paper, a multi-sensor information fusion method based on the factor graph topology is proposed. The global optimum solution is factorized according to the chain structure of the factor graph. It can convert the fusion matter into connecting factors in the factor graph, which allows for a more general form of the conditional probability density. It can convert the fusion matter into connecting factors defined by these measurements to the factor graph without considering the relationship between the sensor update frequency and the fusion period. According to the factor graph theory, the maximum a posteriori probability density is derived in the form of the factor node and the variable node. By selecting the appropriate cost function of these nodes, the optimal navigation solution of the system can be calculated. An experimental MUAV system has been built, and some experiments have been performed to prove the effectiveness of the proposed method.

## Figures and Tables

**Table 1:** Parameters of navigation sensors.

Sensor	Error	Value	Frequency
IMU	Gyro constant drift	10°/h	50 Hz
	Gyro first-order Markov process	10°/h	
	Gyro white noise measurement	10°/h	
	Accelerometer first-order Markov process	1 × 10^−4^ g	
GPS	Position error noise	10 m, 10 m, 20 m	1 Hz
	Velocity error noise	0.1 m/s, 0.1 m/s, 0.1 m/s	
Magnetometer	Heading error noise	0.2°	20 Hz
Barometer	Height error noise	5 m	10 Hz

**Table 2:** Statistical error contrast between two schemes.

Error Type	Average RMSE in the Position Error (units: m)			Average RMSE in the Velocity Error (units: m/s)		
	Longitude	Latitude	Height	Eastern	Northern	Vertical
Extend Kalman filter	1.212	1.205	0.703	0.141	0.142	0.049
Factor graph filter	1.043	1.035	0.628	0.121	0.115	0.034

**Table 3:** System dynamic parameters.

Type	Parameters Item	Unit
Machine size	608 × 608 × 243	mm
Takeoff weight	950	g
Maximum payload	<580	g
Flight time	15	min

**Table 4:** Statistical error contrast between two filters.

Error Type	RMSE in the Position Error (units: m)			RMSE in the Velocity Error (units: m/s)		
	Longitude	Latitude	Height	Eastern	Northern	Vertical
Extend Kalman filter	1.821	1.451	0.652	0.088	0.086	0.061
Factor graph filter	1.288	1.143	0.519	0.065	0.061	0.049
